# Forelimb EMG-based trigger to control an electronic spinal bridge to enable hindlimb stepping after a complete spinal cord lesion in rats

**DOI:** 10.1186/1743-0003-9-38

**Published:** 2012-06-12

**Authors:** Parag Gad, Jonathan Woodbridge, Igor Lavrov, Hui Zhong, Roland R Roy, Majid Sarrafzadeh, V Reggie Edgerton

**Affiliations:** 1 Biomedical Engineering IDP, University of California, Los Angeles, CA, 90095, USA; 2 Department of Computer Science, University of California, Los Angeles, CA, 90095, USA; 3 Department of Integrative Biology and Physiology, University of California, Los Angeles, CA, 90095, USA; 4 Neurobiology, University of California, Los Angeles, CA, 90095, USA; 5 Brain Research Institute, University of California, Los Angeles, CA, 90095, USA

**Keywords:** Spinal cord injury, Spinal bridge-assisted stepping, EMG detection, Fast Fourier transform

## Abstract

**Background:**

A complete spinal cord transection results in loss of all supraspinal motor control below the level of the injury. The neural circuitry in the lumbosacral spinal cord, however, can generate locomotor patterns in the hindlimbs of rats and cats with the aid of motor training, epidural stimulation and/or administration of monoaminergic agonists. We hypothesized that there are patterns of EMG signals from the forelimbs during quadrupedal locomotion that uniquely represent a signal for the “intent” to step with the hindlimbs. These observations led us to determine whether this type of “indirect” volitional control of stepping can be achieved after a complete spinal cord injury. The objective of this study was to develop an electronic bridge across the lesion of the spinal cord to facilitate hindlimb stepping after a complete mid-thoracic spinal cord injury in adult rats.

**Methods:**

We developed an electronic spinal bridge that can detect specific patterns of EMG activity from the forelimb muscles to initiate electrical-enabling motor control **(**eEmc) of the lumbosacral spinal cord to enable quadrupedal stepping after a complete spinal cord transection in rats. A moving window detection algorithm was implemented in a small microprocessor to detect biceps brachii EMG activity bilaterally that then was used to initiate and terminate epidural stimulation in the lumbosacral spinal cord. We found dominant frequencies of 180–220 Hz in the EMG of the forelimb muscles during active periods, whereas these frequencies were between 0–10 Hz when the muscles were inactive.

**Results and conclusions:**

Once the algorithm was validated to represent kinematically appropriate quadrupedal stepping, we observed that the algorithm could reliably detect, initiate, and facilitate stepping under different pharmacological conditions and at various treadmill speeds.

## Background

Functionally complete spinal cord injury is a severe debilitating condition and leads to paralysis. Numerous approaches have been attempted to recover function after paralysis, e.g., facilitation of axon regeneration including methods to suppress growth inhibitory molecules, modulation of the levels of neurotrophic factors, cell transplantation, and the use of activity-dependent mechanisms [1-4]. These techniques, however, have not resulted in dramatic improvements of motor function after motor complete paralysis. A technique that has shown promise is Brain-Computer Interface. This approach has been successfully developed in integrating activity from the functionally unaffected sites, such as the motor cortex, to control robotic devices or muscle stimulating devices to generate the desired movement in paralyzed muscle groups [5].

Recent *in vivo* studies in rats and cats show that networks of neurons in the lumbosacral region of the spinal cord have an intrinsic capability to generate coordinated rhythmic motor outputs in the hindlimbs [2,6,7]. Several strategies have been tested to tap into these neural circuits and activate them to induce oscillatory motions in the hindlimbs. For example, pharmacologically enabling motor control strategies (fEmc) using serotonergic agonists of 5-HT_1A,2A_ and 5-HT_7_ receptors in combination with epidural stimulation, i.e., electrical-enabling motor control (eEmc) [8-10], have been used to recover considerable function after paralysis. These two interventions combined with the availability of sensory information in real time have been used to induce full weight-bearing stepping in complete spinal rats [10,11]. Gerasimenko et al. [8] have shown that eEmc (at 40 Hz with monopolar stimulation) between the L2 and S1 spinal cord levels facilitates bilateral stepping of spinal rats on a moving treadmill belt. In contrast, the spinal rats did not step when the treadmill was turned on but no eEmc was provided, indicating that the stimulation was necessary for the spinal rats to step.

These observations led us to ask whether ‘indirect’ volitional control of eEmc (by forelimb EMG activity) could be used to facilitate stepping in the hindlimbs and provide a new and stable level of control of motor function in spinal rats. Therefore, the purpose of this study was to determine whether ‘indirect’ volitional control via an electronic spinal bridge could be accomplished. In human subjects, this volitional control could avoid the use of an external switch to activate an electrode array by using EMG signals as occurs during normal locomotion. As importantly the present experiments provide a testbed for development of a Brain-Machine-Spinal Cord Interface (BMSCI) that allows for motor control with minimal conscious attention. In addition, it provides a potential mechanism for exerting finer motor control than could be accomplished using a simple on/off system. To design and test such a ‘Brain-Machine-Spinal Cord Interface’, we used EMG signals from the forelimbs as a trigger to initiate spinal cord stimulation to facilitate movement of the hindlimbs. We used the forelimb EMG because these signals are part of the natural gait cycle in quadrupedal stepping.

The objectives of this study were to develop an effective pattern of step detection from the uninjured forelimb muscles and to use this pattern to control the on/off state of the eEmc of the spinal cord to facilitate quadrupedal stepping under different experimental conditions in spinal rats. We observed that voluntary signals from the forelimbs during stepping can be used as a control mechanism for generating signals to the spinal cord to facilitate hindlimb stepping. The underlying assumption is that when the rat “intends” to step the forelimbs will be activated in a pattern that reflects this “voluntary” intent and that intent will initiate eEmc to facilitate stepping of the hindlimbs.

## Methods

Adult female Sprague–Dawley rats (n=5, ~300 g body weight) were used. Pre- and post-surgical animal care has been described previously [12]. The rats were housed individually with food and water provided ad libitum. All survival surgical procedures were conducted under aseptic conditions and with the rats deeply anesthetized (isoflurane gas administered via facemask as needed). All procedures described below are in accordance with the National Institute of Health Guide for the Care and Use of Laboratory Animals and were approved by the Animal Research Committee at UCLA.

### Experimental design

The rats underwent two separate surgeries. The first surgery was to implant the EMG electrodes. The rats were allowed to recover from this implant surgery for one week and then recordings (pre-transection) were made while the rats stepped on the treadmill. After completing these recordings, the rats underwent a second surgery during which the spinal cord was completely transected at a mid-thoracic level and epidural electrodes were implanted at spinal levels L2 and S1. The rats were allowed to recover for one week and then the training sessions were initiated. Recordings were performed to test the electronic bridge at 5 weeks post-transection. The pre-transection EMG recordings provided a baseline for comparison with the recordings post-transection. All of these procedures are performed routinely in our laboratory [9,13]. Details of each step are given below.

### Head connector implantation

A small incision was made at the midline of the skull. The muscles and fascia were retracted laterally, small grooves were made in the skull with a scalpel, and the skull was dried thoroughly. Two amphenol head connectors with Teflon-coated stainless steel wires (AS632, Cooner Wire, Chatsworth CA) were securely attached to the skull with screws and dental cement as described previously [14,15].

### Intramuscular EMG electrode implantation

Selected hindlimb (tibialis anterior, TA; and soleus, Sol) and forelimb (biceps brachii, BB; and triceps brachii, TB) muscles were implanted bilaterally with EMG recording electrodes as described by Roy et al. [14]. Skin and fascial incisions were made to expose the belly of each muscle. Two wires extending from the skull-mounted connector were routed subcutaneously to each muscle. The wires were inserted into the muscle belly using a 23-gauge needle and a small notch (~0.5-1.0 mm) was removed from the insulation of each wire to expose the conductor and form the electrodes. The wires were secured in the belly of the muscle via a suture on the wire at its entrance into and exit from the muscle belly. The wires were looped at the entrance site to provide stress relief. The proper placement of the electrodes was verified during the surgery by stimulating through the head connector and post-mortem via dissection.

### Spinal cord transection

A partial laminectomy was performed at the T8-T9 vertebral level and a longitudinal cut was made in the dura to expose the spinal cord. A complete spinal cord transection to include the dura was performed at approximately the T8 spinal level using microscissors. Two surgeons verified the completeness of the transection by lifting the cut ends of the spinal cord and passing a glass probe through the lesion site. Gel foam was inserted into the gap created by the transection as a coagulant and to separate the cut ends of the spinal cord.

### Epidural electrode implantation

Epidural electrodes were coiled and left in the back region of the animal after the EMG surgery. The epidural electrodes were implanted during the second surgery. Partial laminectomies were performed to expose the spinal cord at spinal levels L2 and S1. Two Teflon-coated stainless steel wires from the head connector were passed under the spinous processes and above the dura mater of the remaining vertebrae between the partial laminectomy sites. After removing a small portion (~1 mm notch) of the Teflon coating and exposing the wire on the surface facing the spinal cord, the electrodes were sutured to the dura mater at the midline of the spinal cord above and below the electrode sites using 8.0 Ethilon suture **(**Ethicon, New Brunswick, NJ**)**. A common ground (indifferent) wire (~1 cm of the Teflon coating removed distally) was inserted subcutaneously in the mid-back region. All wires were coiled in the back region to provide stress relief.

All incision areas were irrigated liberally with warm, sterile saline. All surgical sites were closed in layers, i.e., muscle and connective tissue layers with Vicryl (Ethicon, New Brunswick, NJ) and the skin incision on the back with Ethilon and in the limbs with Vicryl. All closed incision sites were cleansed thoroughly with saline solution. Analgesia was provided by buprenex (0.5–1.0 mg/kg, s.c., 3 times/day). The analgesics were initiated before completion of the surgery and continued for a minimum of 2 days. The rats were allowed to fully recover from anesthesia in an incubator. The rats were housed individually, and the bladders of the spinal rats were expressed manually 3 times/day for the first 2 weeks after surgery and 2 times per day thereafter. The hindlimbs of the spinal rats were moved passively through a full range of motion once per day to maintain joint mobility. All of these animal care procedures have been described in detail previously [12].

### Stimulation and training procedures

All rats were trained to step quadrupedally using a body weight support system under the influence of quipazine administration (0.3 mg/kg, i.p.) and eEmc (40 Hz, between L2 and S1 with the current flowing from L2 to S1) [9,13,16]. The maximum stimulation voltage used was 3 V and the stimulation intensity was modulated to produce maximum stepping performance. The rats stepped on a specially designed motor-driven rodent treadmill. The treadmill belt had an anti-slip material that minimized slipping while stepping. The rats were trained using a body weight support system: the rats were suspended in a jacket such that all four limbs were in contact with the treadmill and that there was enough room for all 4 limbs to carry out the swing and stance phases of the step cycle. This was a critical component of the design as it was important to engage the forelimbs in stepping to produce robust, high quality EMG signals from the forelimb muscles.

### Testing procedures

Pre-transection the rats were stepped quadrupedally on the treadmill at varying speeds (13.5 to 21 cm/s) without the use of the body weight support system. These baseline recordings were compared to post-transection recordings. Five days post-transection, the rats were fitted with a jacket and secured to the body weight system for a period of 2–3 min initially and then the time was progressively increased to about 10 min by day 7. This was an acclimation period and the rats were not stepped during this period. Training began one-week post-transection. Stepping ability was tested once a week pre-quipazine and 15 minutes post-quipazine administration. Quipazine (a serotoninergic agonist) administered intraperitoneally (0.3 mg/kg) has been shown to improve stepping performance of spinal animals when receiving eEmc [8,16]. We used quipazine administration in the present study to produce robust stepping in the spinal rats. Kinematics and EMG data were collected on a weekly basis from all rats. The algorithm for detection of forelimb stepping was based on these data.

### Data acquisition and post-processing

**Figure 1:**
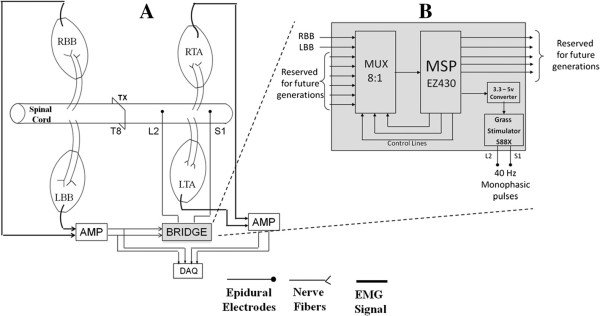
**Electronic bridge schematic and circuitry.A**) Schematic diagram showing the design of the electronic bridge. EMG signals from the right biceps brachii (RBB) and left BB (LBB) are sent to the bridge. Forelimb stepping is detected at the bridge that then generates electrical pulses (40 Hz monopolar) in the lumbosacral spinal cord to generate stepping in the hindlimbs. EMG from the forelimb and hindlimb muscles are amplified and stored using the DAQ. **B**) Expanded view of the electronic bridge circuitry.

EMG recordings from the forelimb and hindlimb muscles were band-pass filtered (1 Hz to 1 KHz), amplified using an A-M Systems Model 1700 differential AC amplifier (A-M Systems, Carlsborg, WA), and sampled at a frequency of 10 KHz using a custom data acquisition program written in the LabView development environment (National Instruments, Austin, TX) as described previously [9]. The EMG signals from the forelimbs also were sent to the TI MSP430 where they went through a ADC to be processed for step detection. Raw analog EMG signals were collected, filtered, digitized, and processed in real time by the microprocessor (Figure 1).

### Kinematics recording parameters

Additional file 1: Video recordings of the hip, knee, ankle, shoulder, elbow, and wrist joints were obtained to study the segmental and joint angle kinematics during stepping. A four-camera system was calibrated and then used to track reflective markers placed on bony landmarks on the iliac crest, greater trochanter, lateral condyle, lateral malleolus, the distal end of the fifth metatarsal of both hindlimbs, and the head of the humerus, olecranon process, radial process, and tips of the paw of both forelimbs. The video footage was processed using SIMI Motion analysis software (SIMI, Unterschleissheim, Germany) to produce the 3-D reconstruction of the hindlimb and forelimb movements, as well as the 2-D ball-and-stick diagrams and hindlimb trajectory plots [9]. The 3-D coordinates for a given marker were calculated using a triangulation procedure that partially accounts for the movement of the skin. This is a technique that has been used successfully and implemented in many labs and has the precision necessary for the present study.

### Electronic bridge schematic

Figure 1A shows the schematic of the electronic bridge and the experimental setup. The EMG signals from the forelimb muscles were fed to the electronic bridge. The output of the electronic bridge was connected to wire electrodes implanted at specific levels on the spinal cord. Figure 1B shows an expanded view of the electronic bridge. We used an 8:1 MUX (MAX14752, Maxim) that is controlled by a microcontroller (MSP EZ430, Texas Instruments). The electronic bridge has 2 input channels (RBB and LBB) and one output channel (Stim 1) while 5 other channels are reserved for future use. The EZ430 contains 10 I/O channels and 5 of these channels are used for this system (3 control lines, 1 input and 1 output).

The MSP EZ430 has an inbuilt 10-bit ADC with a maximum sampling rate of 200 kHz. It consists of 10 general-purpose analog I/O lines that allow the design to be flexible along with potential additions to the design in the future. The MSP EZ430 is powered by a DC source that provides a maximum output of 3.3 V. The minimum voltage required to trigger the Grass stimulator is 5 V. The 3.3 V output is converted to a 5 V output using a simple NE555 timer that is synchronized with the output of the MSP EZ430 to provide 40 Hz pulses.

### EMG detection techniques

**Figure 2:**
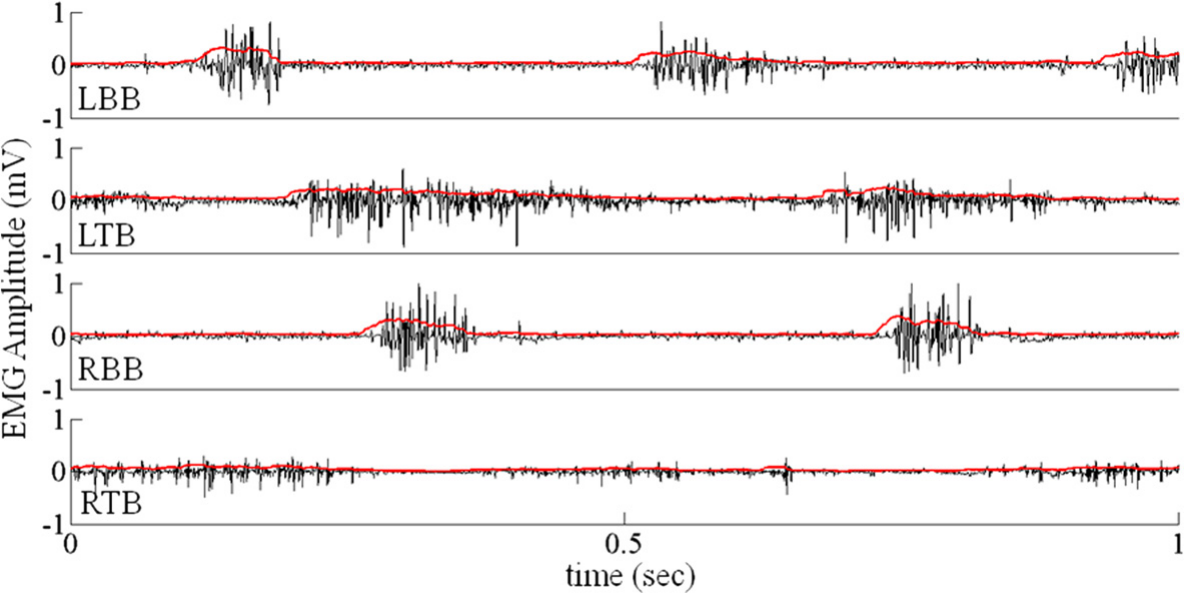
**EMG detection strategies – First Generation.** Sequence of strategies used to detect stepping in the first generation included: i) calculating the linear envelope (shown by the red lines) of each signal using a moving window standard deviation technique, ii) reciprocal activity between the BBs and TBs bilaterally, and iii) a constant phase difference between the left and right forelimbs.

**Figure 3:**
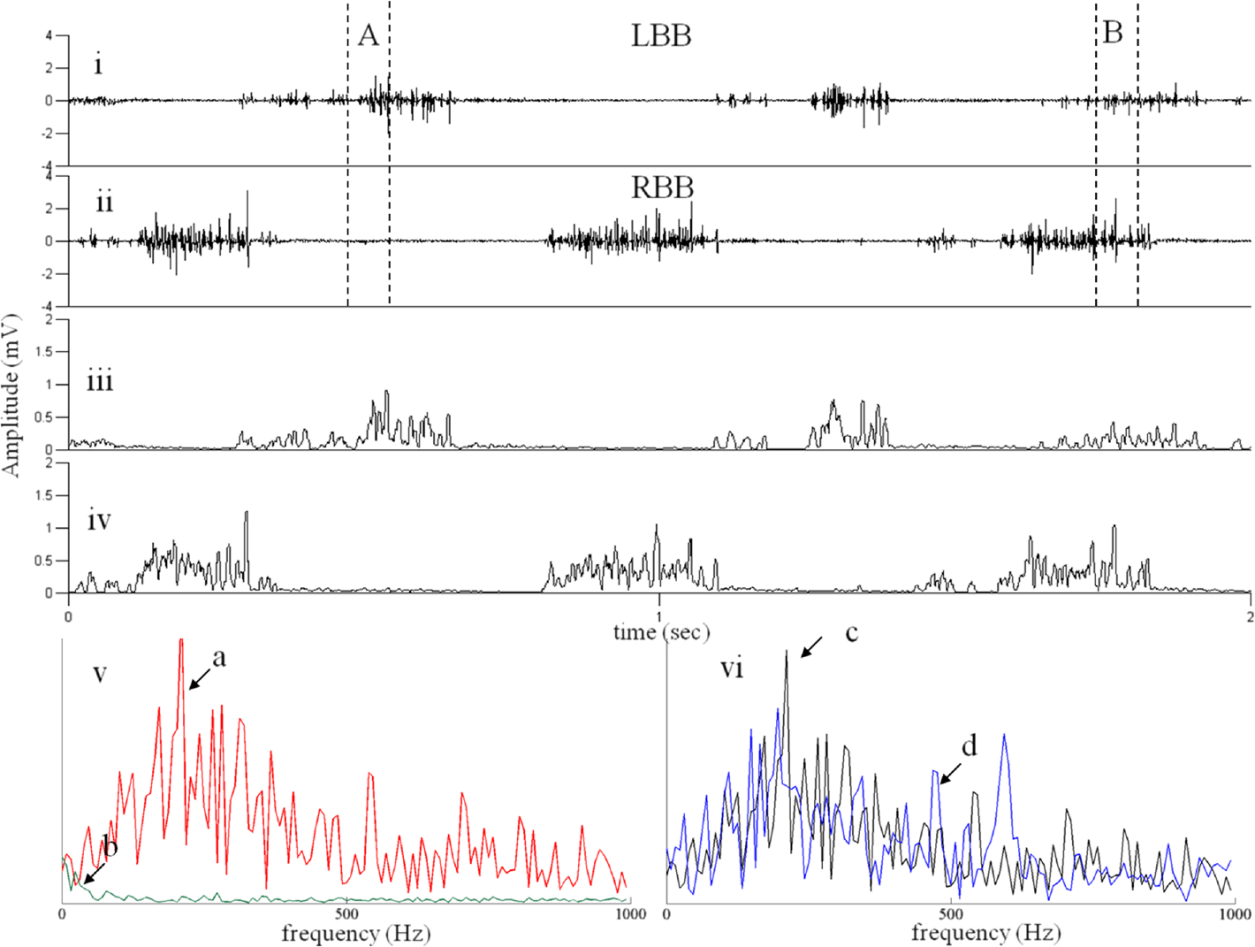
**EMG detection strategies – Second Generation.** Raw EMG (i and ii), moving window standard deviation used in the first generation (iii and iv) and frequency spectrum used in the second generation (v) for the LBB (red) and RBB (green) for region marked by A and (vi) for the LBB (black) and RBB (blue) for region marked by B. during stepping. In v the frequency spectrum of the signals shown in A when there is reciprocal activity and in vi the frequency spectrum shown in B when there is co-activation.

Two strategies were attempted for detecting stepping in the forelimbs. The first attempt at an electronic bridge involved the detection of the reciprocity of the EMG activity of the BB and TB (Figure 2) using a moving window standard deviation technique. We calculated the standard deviation using a window of 20 consecutive data points and assigned the calculated value to the first data point. This procedure was repeated by moving the window across the length of the signal. The resultant plot of the standard deviation calculation forms a positive linear envelope around the raw EMG signals (Figures 2 and 3iii and iv). Using the resultant signal we applied an optimum threshold to digitize the signals bilaterally from the BB and TB. Our objective was to achieve antagonistic action in the BB and TB and a finite phase difference between the left and right sides. This technique was effective and was successful in detecting stepping, but was limited in two ways: 1) it was dependent on the burst duration of all muscles involved; and 2) different thresholds were needed for each muscle. These thresholds vary from animal to animal and change over time. Accounting for these changes requires human intervention.

**Figure 4:**
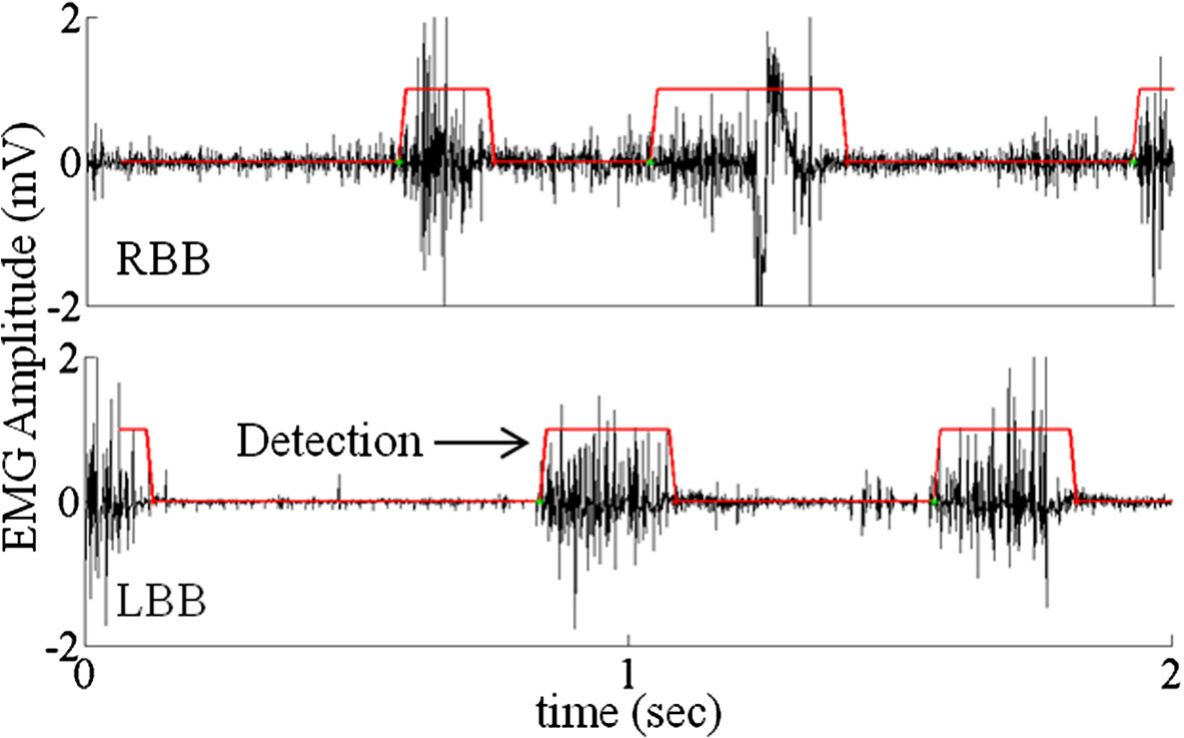
**Detection of EMG activity using the FFT technique.** EMG activity from RBB and LBB superimposed with activity detection (shown by the red lines) using the frequency analysis algorithm (also see Figure 3v and vi). This algorithm detects the start and end of each EMG burst. These data show examples of a period during which there is continuous stimulation through the bridge.

**Figure 5:**
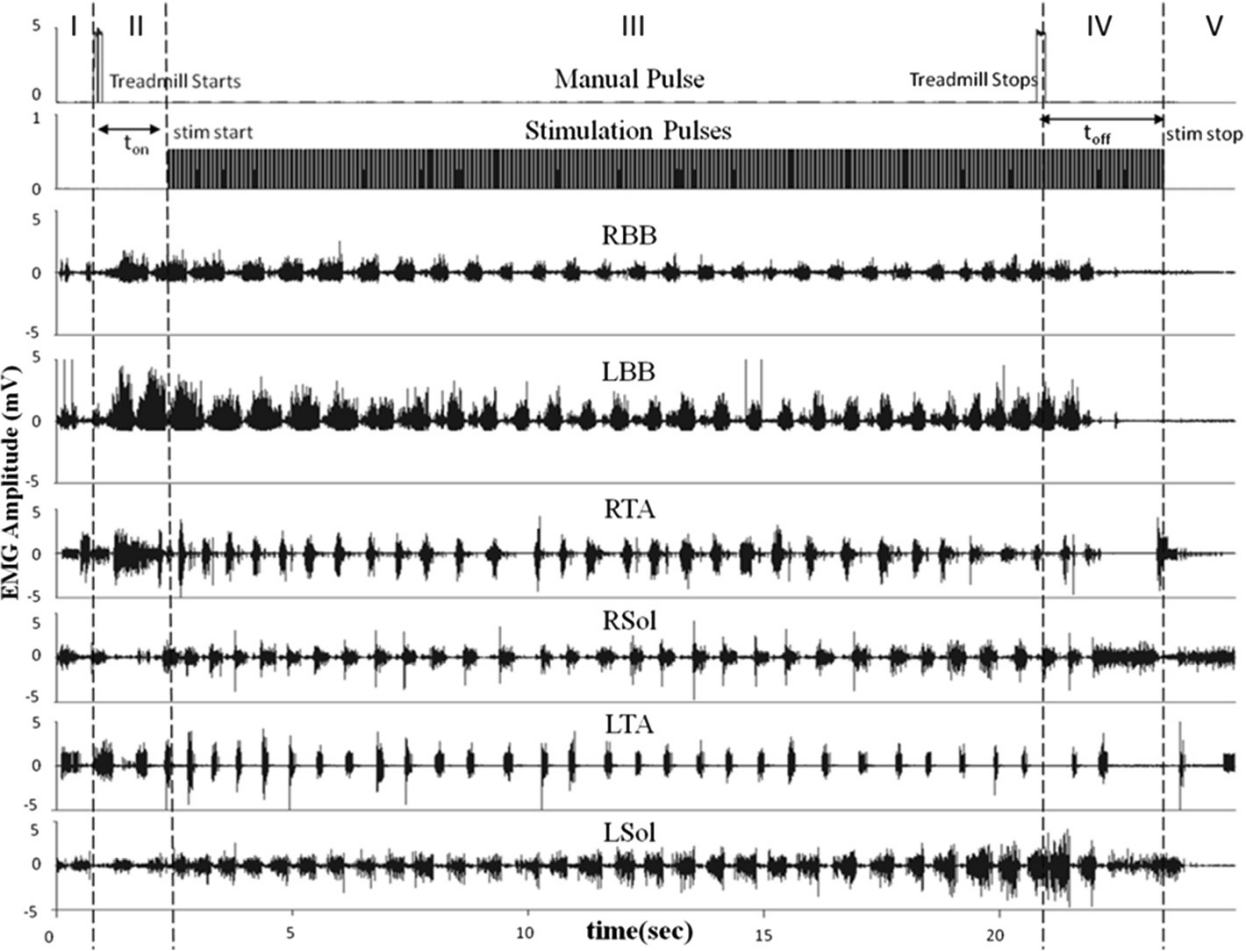
**EMG recorded during stimulation through the second generation electronic bridge.** Manual pulse indicates the turning on (first manual pulse at the beginning of Phase II) and turning off (second manual pulse at the end of Phase III) of the treadmill. Alternating RBB and LBB EMGs are read by the microcontroller to detect forelimb stepping. Electrical pulses are generated in the lumbosacral spinal cord resulting in hindlimb stepping. Alternating EMG bursts in the tibialis anterior (TA, ankle flexor) and soleus (Sol, ankle extensor) muscles of each leg indicate rhythmic movements of the hindlimbs.

Due to the problems faced by the first technique, we developed a second technique that would require little or no need for setting thresholds for detection of activity and calibration of the system. Such a step detection algorithm must exhibit low complexity with both memory and processing. Calibrating an EMG system can be an extremely time-consuming process. Another disadvantage is that, EMG must be calibrated often for each animal as well as physiological changes occur during recovery from an injury. To avoid requirements of calibration, this electronic bridge step-detection algorithm converts EMG signals to the frequency domain using a Fourier transform. We found the absolute value of each frequency to determine the overall frequency power curve (Figure 3v and vi). Exact timing of movements, however, is required to efficiently detect stepping patterns as well as to stimulate the rats properly. For this reason, we computed the 128-point Fourier transform at each point in time. In this scenario, a sliding window discrete Fourier transform (DFT) as described by Jacobsen and Lyons [17] is more computationally efficient than using the standard fast Fourier transform (FFT) as described by Cooley et al. [18]. The following underlying principles are followed in the step detection algorithm: 1) calculation of a 128-point sliding window DFT at each time point; 2) rhythmic activity in both BBs during stepping; and 3) an alternating phase difference between the left and right BBs. An alternating phase is present when the EMG burst in one limb begins after 30% of the contralateral cycle period has been completed. Once these three conditions are met, a counter is initialized and continued to the completion of two steps at which time the signals to initiate eEmc are sent to the spinal cord (Figures 4 and 5).

Figure 4 represents the result of the frequency analysis technique. We observed that the RBB has higher baseline noise as compared to the LBB. Using the frequency response we were able to successfully define the bursts even in the case of large baseline noise. The use of the second technique allowed us to detect stepping based on the variation in the EMG frequency spectrum between the active phase and the inactive phase independent of the amplitude of the EMG signals.

We tested the stability of the detection algorithm of the bridge 5 weeks post-transection at treadmill speeds of 13.5 and 21 cm/s and with and without quipazine administration. This enabled us to test the stability of the detection algorithm under different conditions (better hindlimb stepping with than without quipazine administration at both speeds). All rats were tested for a total of 5 trials under each condition and the 3 best trials were chosen for analysis. The efficiency of the electronic bridge was tested by measuring t_on_ and t_off_ for the various test conditions. t_on_ was defined as the time between the treadmill turning on and the stimulation turning on (time needed for the bridge to detect stepping). t_off_ was defined as the time between the treadmill turning off and the stimulation turning off (time needed for the bridge to detect the end of stepping).

We also tested the stepping ability of the rats at two treadmill speeds with and without quipazine with direct stimulation (without the electronic bridge) where the experimenter turned on the stimulation manually. This enabled us to compare the stepping ability with and without the electronic bridge.

### Algorithm validation

Having identified three criteria based on FFT analysis of EMG as described above it was also necessary to validate whether these criteria resulted in kinematics characteristics associated with and without forelimb weight bearing. This validation was performed with respect to the speed of locomotion and with different pharmacological interventions. The single kinematics criteria reflecting successful stepping was a change in elbow angle bilaterally of at least 50° for five consecutive steps.

### Statistical analyses

All data are reported as mean±SEM. Statistically significant differences were determined using a two-way (t_on_ and t_off_ under 4 experimental conditions) analysis of variance (ANOVA). The criterion level for determination of statistical significance was set at P < 0.05 for all computations.

## Results

### Voluntarily induced stepping

**Figure 6:**
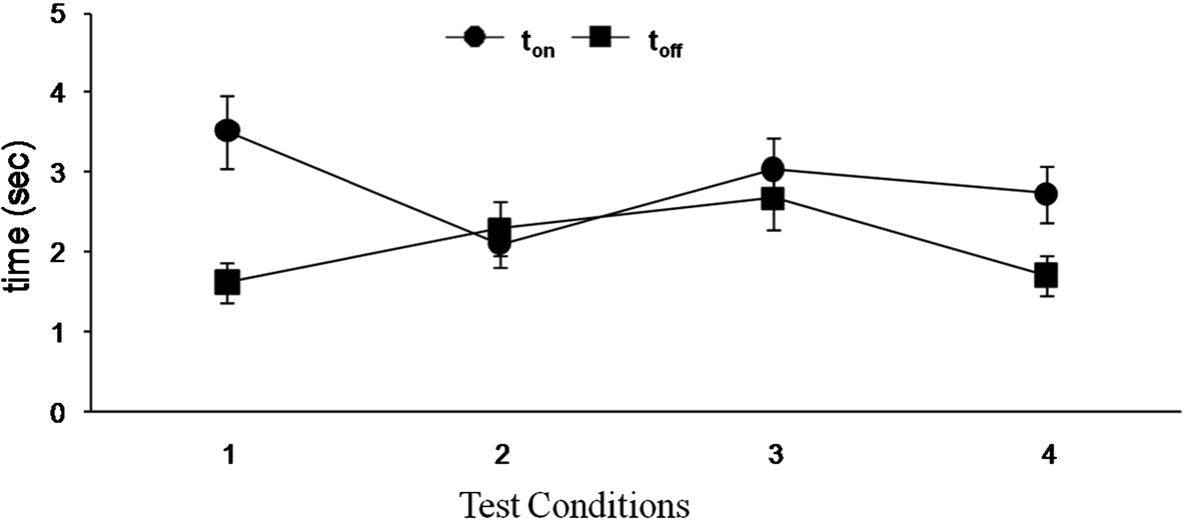
**Response times for the electronic bridge under four test conditions.** Mean response times (mean ± SEM, n = 5 rats) calculated by the algorithm to start (t_on_) and stop (t_off_) stimulation under four test conditions of quadrupedal stepping on a treadmill: 1) 13.5 cm/s pre-quipazine; 2) 13.5 cm/s post-quipazine; 3) 21 cm/s pre-quipazine; and 4) 21 cm/s post-quipazine administration. Note that there are no significant differences between t_on_ and t_off_ for any test condition or across all test conditions.

We assessed the effectiveness of the algorithm for the electronic bridge when the rats were stepping consistently at 13.5 and 21 cm/s on a treadmill. Figure 4 shows a typical EMG recording using the electronic bridge. The EMG recording was divided into 5 phases to better understand the EMG of the forelimbs and hindlimbs and the state of the stimulation pulses. Phase I: prior to the first manual pulse and with the treadmill turned off, there is some random motion in the forelimbs that is not detected by the electronic bridge as stepping. Phase II: the treadmill is turned on and moving at a constant speed, resulting in forelimb stepping. Alternating EMG in the forelimb muscles is detected and triggers a counter that, on reaching a pre-determined threshold (two steps), sends pulses to the spinal cord at a preset frequency at the end of Phase II. The counter is designed to avoid false positives. Phase III: the treadmill is moving at the same speed and the EMG from the forelimb muscles continues to be detected. eEmc results in oscillatory weight-bearing movements in the hindlimbs. Phase IV: the treadmill is turned off (second manual pulse) and movements in the forelimbs are reduced progressively. In this phase, the detection of the forelimb muscle EMG ends, and this triggers a downcounter. Once the downcounter reaches zero, stimulation stops. In this phase, even though the treadmill is turned off some motion in the hindlimbs remains due to a residual effect of stimulation. Phase V: this phase is identical to Phase I where the microprocessor is looking for detection of EMG in the forelimb muscles. There were no significant differences for either t_on_ or t_off_ across the 4 conditions tested at the P = 0.05 level (Figure 6). The lack of a difference in t_on_ and t_off_ across the four conditions demonstrates the robustness of the algorithm.

### Does the electronic bridge have a detrimental effect on stepping performance?

**Figure 7:**
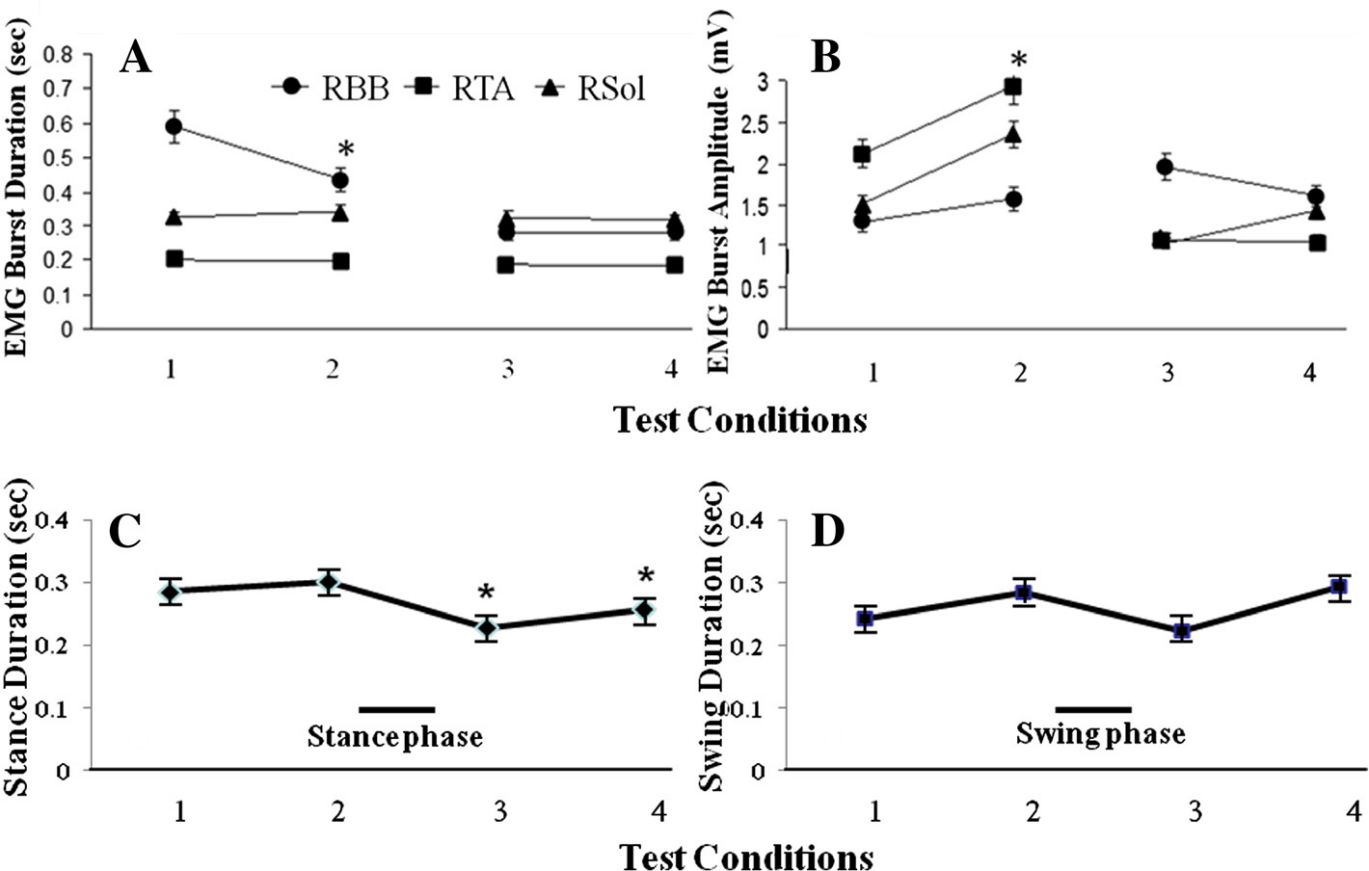
**EMG responses during four test conditions.A** and **B**: Mean EMG burst durations and amplitudes (mean ± SEM, n = 5 rats) for hindlimb and forelimb muscles during stimulation via the bridge under the same four conditions tested in Figure 6. ^*^, RBB in test condition 2 is significantly different from test condition 1. **C** and **D**: mean duration of the stance phase and swing phase (mean ± SEM, n = 5 rats) during stimulation via the bridge under the same four conditions tested in Figure 6. *, duration of the stance phase is significantly lower for condition 3 compared to condition 1 and for case 4 compared to case 2.

**Figure 8:**
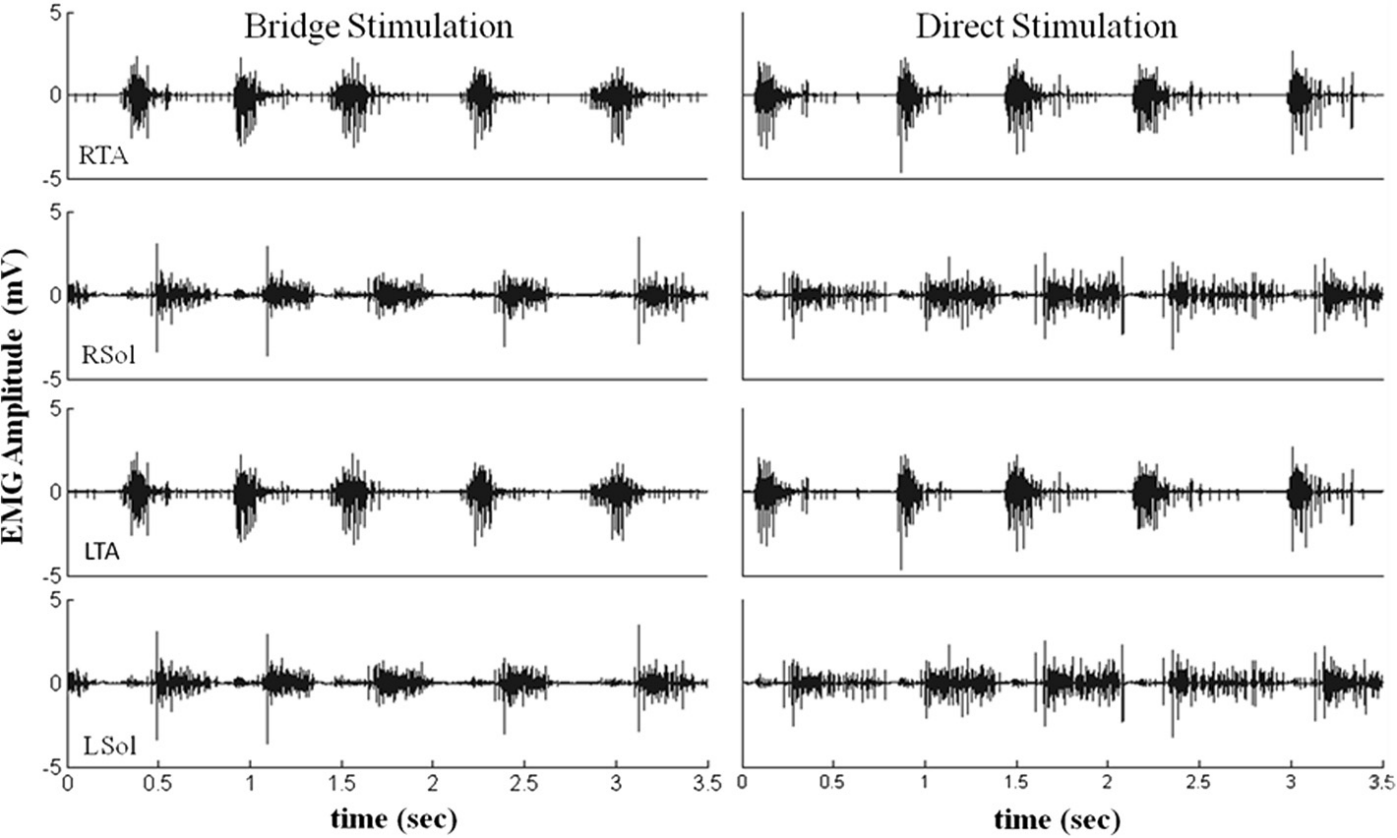
**Stepping during electronic bridge stimulation vs. direct stimulation.** Raw EMG activity from the hindlimb muscles bilaterally during quadrupedal stepping on a treadmill at 13.5 cm/s with stimulation via the bridge and with direct stimulation (stimulation without the use of the bridge).

We used quipazine administration in the present study to produce robust quadrupedal stepping in the spinal rats as reported previously for bipedal stepping [8,16]. The mean EMG burst durations and amplitudes for selected forelimb and hindlimb muscles during the 4 conditions tested while being stimulated via the bridge are shown in Figure 7. There was a significant decrease in the RBB EMG burst duration between pre- and post-quipazine stepping at 13.5 cm/s. Regardless of this difference, the response time of the electronic bridge was similar with and without quipazine administration, i.e., quipazine did not affect the detection protocol. The EMG burst characteristics of the hindlimb muscles were not different with direct stimulation (no electronic bridge) and with the electronic bridge, indicating that the electronic bridge does not affect the stepping performance (Figure 8). With increasing treadmill speeds, the ankle flexors had a relatively constant burst duration, whereas the ankle extensors had a shorter burst duration (Figure 7), consistent with that reported previously in control adult rats [14].

### Algorithm validation: Determining the detection threshold for stepping

**Figure 9:**
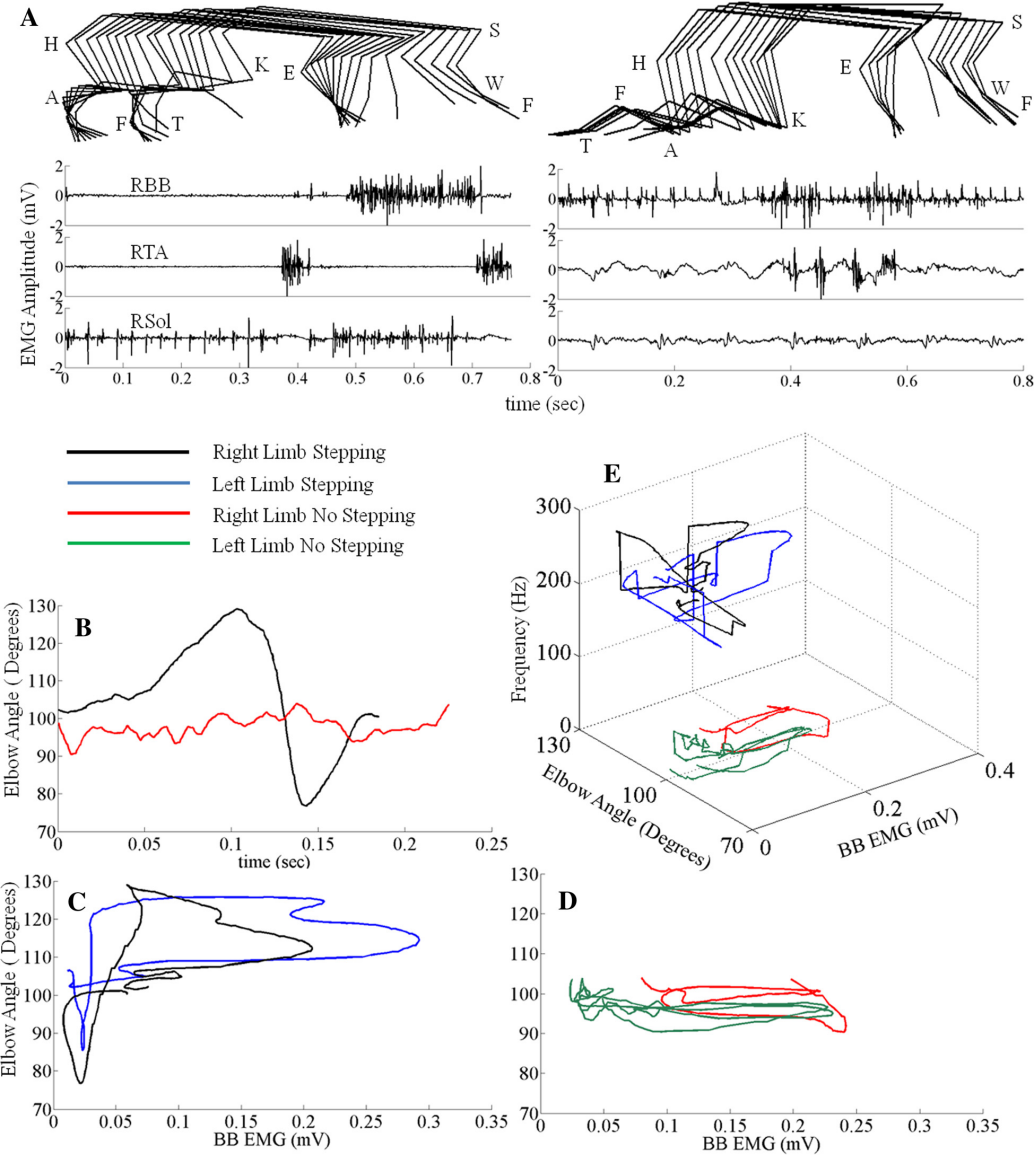
**Algorithm validation using offline testing.** EMG and kinematics responses when the forelimbs were stepping or not stepping. (**A**) 2-D stick diagrams (50 ms between sticks) of the limbs observed when the forelimbs were stepping or not stepping with the corresponding EMG. (B) Changes in the elbow angle for the data shown in **(A)**. (**C**) Scatterplot between the linear envelope of the BB EMG and the elbow angle during stepping. (**D**) Scatterplot between the linear envelope of the BB EMG and the elbow angle when the forelimbs were not stepping. (**E**) 3-D plot representing the elbow angle, BB EMG, and peak frequency (see Figure 3v and vi and Figure 4).

We compared the kinematics and the effectiveness of the detection algorithm in the presence and the absence of stepping (Figure 9A). Using the forelimbs as a reference, the step cycle began when the forelimb touched the treadmill belt and ended when it touched the treadmill belt again. The kinematics were derived from the angles of the elbow during stepping using 3-D coordinates. Figure 9B shows the variation in the angle of the right elbow in the same step cycle as shown in Figure 9A. The black trace represents the angle of the elbow when the forelimbs were stepping, whereas the red trace represents the angle of the elbow when there was no forelimb stepping. Figure 9C and D show the scatterplots between the amplitude of the BB EMG envelope and the elbow angle for the same data shown in Figure 9A and B. BB EMG amplitudes were minimal and the elbow angle decreased during the stance phase (when the paw was on the treadmill), whereas the BB EMG amplitude increased and the elbow angle increased during the swing phase (when the paw was off the treadmill). The relationship between the elbow angle and the BB EMG frequency is illustrated in Figure 9E, demonstrating a clear dichotomy of the presence of stepping vs. no stepping based the frequency spectrum.

## Discussion

We have developed a BMSCI having a pattern recognition algorithm that can use EMG from the forelimb muscles to trigger the initiation and termination of the stimulation of the spinal cord below the level of a complete spinal cord injury. This algorithm (second generation) detects stepping with little or no calibration and thus provides an advantage over the system (first generation) we tested initially that needs constant monitoring. Our logic for using the EMG signals from the forelimb muscles as a trigger was that these signals reflect the "intent to step" quadrupedally. The idea was to use naturally generated EMG signals from the forelimbs to control an electronic bridge that would facilitate hindlimb stepping in spinal rats. Our results from multiple test conditions demonstrate that this system is capable of adapting to different pharmacological and stepping conditions. Results from the first generation showed us that it is possible to develop a real time EMG detection system on the MSP430, but this generation was cumbersome to run due to calibration of the multiple channels and the variability among animals. The technique used in the second generation allowed us to reduce the calibration and to accommodate the variability among animals. Given the variability in the conditions under which the step detection algorithm was tested on the MSP430, the time taken to detect the beginning of stimulation (t_on_) and the time taken to detect the stopping of stimulation (t_off_) had about the same consistency and demonstrated the algorithm's ability to detect EMG signals and to adapt to different conditions and different animals (Figure 6).

To further enhance the utility of spinal cord stimulation, the control system must go beyond an on/off control as shown in the present experiments. In paraplegic humans any set of muscles that can be voluntarily controlled could be used as a trigger. Generating and controlling the EMG in muscles such as the deltoid or pectoralis to control prosthetic devices has been shown to be feasible in humans [19]. Harkema et al. [20] demonstrated in a single human subject the importance of varying stimulation parameters in obtaining the most effective standing and voluntary control of the legs in a subject with a motor complete injury. Similarly, Ichiyama et al. [15] reported that there was variation in the required stimulation voltage at different time points after injury: the stimulation intensity needed to generate locomotor activity in spinal rats was 3.39 ± 0.2 V at 2 weeks and 7.55 ± 1.2 V at 4 weeks post-ST using 40 Hz stimulation monopolar stimulation at L2.

Dutta et al. [21] reported a system to detect EMG during stepping to trigger muscles in the contralateral limb to initiate stepping in human patients with an incomplete spinal cord injury. A training data set was derived from patients with a switch-based functional electrical stimulation system to assist the patients in stepping and a feature extraction was performed on the EMG. A threshold-based binary classifier was trained to distinguish a set of feature templates in the EMG linear envelopes indicating the intention to trigger the next step. These authors report that during online testing the intention to trigger the next step was detected using analyses between features extracted from real time EMG linear envelopes and feature templates derived from the training data set.

The ultimate objective of the present study was to use EMG from multiple muscles in the forelimb to detect stepping and fine variations in forelimb and hindlimb stepping and to modulate the stimulation of the spinal cord to generate optimum stepping movements in the hindlimbs. We also have developed a simulated test bench in MATLAB (Mathworks) to test the EMG signals for offline step detection. This testbench is an exact replica of the algorithm run on the MSP430. Using this simulated test bench, we can validate the recordings seen in real time as well as test the detection algorithm at different time points during recovery after an injury. Further simulations could be used to develop a ‘self-learning’ or ‘threshold-adjusting’ algorithm that could be animal specific as well as being applicable for different stages of recovery after an injury.

## Conclusions

We have developed a novel technique for detecting stepping of the forelimbs and neuromodulating the spinal circuitry in real time to control hindlimb movements in rats with complete paralysis. This detection algorithm can accommodate the variations in EMG amplitudes that normally occur during spontaneous functional recovery after a spinal cord injury. This neuromodulatory approach also is likely to have the potential to improve the control of movements in other neuromotor disorders, such as stroke and Parkinson Disease.

## Abbreviations

BMSCI: Brain-Machine-Spinal Cord Interface; DFT: Discrete Fourier Transform; EMG: Electromyography; ES: Epidural Stimulation; LBB: Left Biceps Brachii; FFT: Fast Fourier Transform; LSol: Left Soleus; LTA: Left Tibialis Anterior; LTB: Left Triceps Brachii; RBB: Right Biceps Brachii; RSol: Right Soleus; RTA: Right Tibialis Anterior; RTB: Right Triceps Brachii.

## Competing interests

The authors have no competing interest.

## Authors' contributions

PG and JW worked on developing the step detection algorithm. PG and IL designed the study, collected and analyzed the data. HZ and RRR performed the surgeries. PG, IL, RRR, and VRE worked on writing the manuscript. All authors read and approved the final manuscript.

## Supplementary Material

Addtitional file 1 **The video file demonstrates a spinal rat stepping quadrupedally on a treadmill at 13.5 cm/s under the influence of eEmc (40 Hz between L2 and S1 spinal levels) and quipazine (0.3 mg/kg, i.p.).** The file can be viewed using any media player such as vlc or windows media player. Click here for file
